# Meta-analysis of single agents in the chemotherapy of NSCLC: what do we want to know?

**DOI:** 10.1054/bjoc.2000.1740

**Published:** 2001-05

**Authors:** A Bahl, S Falk

**Affiliations:** Bristol Haematology and Oncology Centre, Horfield Rd, Bristol BS2 8ED, UK


British Journal of Cancer (2001) 84(9), 1143–1145
© 2001 Cancer Research Campaign
doi: 10.1054/ bjoc.2001.1740, available online at http://www.idealibrary.com on IDEAL®

Editorial

### Meta-analysis of single agents in the chemotherapy of NSCLC: what do we want to know?

**A Bahl and S Falk**

Bristol Haematology and Oncology Centre, Horfield Rd, Bristol BS2 8ED, UK

Evidence has accrued in the last decade that chemotherapy is an effective treatment modality in the management of patients with advanced non-small cell lung cancer (NSCLC) (Non-small Cell Lung Cancer Collaborative Group, 1995). Clinical benefit is evidenced from phase III randomized trials showing survival, symptomatic and quality of life benefits (Non-small Cell Lung Cancer Collaborative Group, 1995; Cullen et al, 1999; Anderson et al, 2000). The optimal chemotherapeutic regimen and the magnitude of benefit in day-to-day clinical practice however remains controversial.

For the large number of patients diagnosed with advanced NSCLC, improvements in systemic therapy offer the only realistic possibility of increasing survival, and probably also local control, which even with surgical intervention, with or without radiation therapy, in stage IIIA disease remains poor (Rosell et al, 1994; Roth et al, 1994). The problem is that to date relatively few active systemic agents have been identified in this context. The survival benefit with chemotherapy demonstrated by meta-analysis in advanced NSCLC was obtained primarily with cisplatin, often in combination with ifosfamide, vindesine and mitomycin C (MMC). The NSCLCCG meta-analysis showed a 27% reduction in the risk of death equivalent to an improvement in survival of 10% at 1 year and an increase in median survival of 6 weeks with cisplatin-based chemotherapy.

Although widely incorporated into combination doublet therapies most commonly with cisplatin or carboplatin, the extent of improvement in outcome with the newer second generation drugs introduced in the last decade including gemcitabine, paclitaxel, docetaxel, vinorelbine and irinotecan has yet to be clearly defined. Early optimism with consistently high response rates for each agent and in combination (Table 1) has not been translated into dramatic survival benefits in advanced NSCLC. In spite of large studies neither has any new combination clearly established itself as a consistently superior reference therapy (Bonomi et al, 2000). Differences in clinical trial populations with varying proportions of locally advanced (stage IIIB) and metastatic (stage IV) NSCLC seems to account for more variation in survival as a principal outcome measure than differences in dosage and/or drug schedules.

During the 1970s single agents with low activity were combined in the hope of attaining a higher response rate and increased survival. Initial enthusiasm for these drug combinations was tempered by an inability to demonstrate a survival benefit in phase III randomized trials. Indeed no randomized trial of first generation agents has in isolation shown a survival advantage for a combination over single agent cisplatin, although response rates may be increased by combination therapy.

*Correspondence to:* S Falk

In this issue Sculier and colleagues have performed a meta-analysis of the role of Mitomycin C (MMC) (Sculier et al, 2001). At present combinations of cisplatin and MMC with either vinblastine or ifosfamide are popular schedules particularly in the UK. Sculier et al report that MMC is associated with a 25% objective response rate when administered as single-agent first-line chemotherapy in advanced NSCLC, but does not improve survival when used in combination with other first generation active cytostatic agents like cisplatin, vindesine and vinblastine. When a large study using a comparison of a probably inadequate dose of vindesine (3 $mg/m^2$ every 2 weeks or 3 $mg/m^2$ weekly for 5 weeks followed by a dose every 2 weeks) is excluded (Luedke et al, 1990) neither does the meta-analysis indicate an improvement in response rate. Sculier et al conclude that MMC should not be used anymore in combination with the first generation active cytostatic agents as it does not improve survival in this setting. They suggest that the role of MMC as salvage chemotherapy or in combination with the second generation active drugs requires further study, owing to the paucity of studies available for analysis.

Inevitably the new agents will shortly be submitted to meta-analysis for possible survival benefits over competitors. Perhaps the major message from this work should be first, a concentration of further discussion about what the relevant end-points are for study with existing agents. Secondly the data should encourage consistency of clinical trial design, particularly in terms of eligibility criteria, and data reporting in NSCLC.

**Table 1** Second generation agents in NSCLC

| Drug | Range of overall response rate (%) | Mean overall response rate (%) |
|---|---|---|
| Gencitabine | 19–23 | 22 |
| Irinotecan | 0–34 | 26 |
| Vinorelbine | 8–36 | 22 |
| Paclitaxel | 22–42 | 28 |
| Docetaxel | 23–39 | 31 |

**Some second generation agents in combination in NSCLC**

| Drugs | Range of overall response rate(%) |
|---|---|
| Gencitabine + Cisplatin | 58–60 |
| Paclitaxel + Carboplatin | 27–63 |
| Cisplatin + Irinotecan | 49–54 |
| Cisplatin + Vinorelbine | 26–52 |
| Paclitaxel + Cisplatin | 31–47 |

Derived and adapted from Bunn, 1996.

**Table 2** Single agent response rates in NSCLC

| Drug | Range of overall response rate (%) | Mean overall response rate (%) |
|---|---|---|
| Ifosfamide | 7–32 | 26 |
| Cisplatin | 6–32 | 20 |
| Mitomycin C | 9–40 | 20 |
| Vindesine | 6–31 | 17 |
| Doxorubicin | 6–38 | 13 |
| Etoposide | 3–21 | 11 |
| Methotrexate | 0–26 | 10 |
| Cyclophosphamide | 4–42 | 8 |

From Bakowski and Crouch, 1983.

Whilst survival is unquestionably the hardest and most important primary end-point of cancer therapies, when given with curative intent, other end-points become of increasing significance when there is likely to be no major difference in survival. When treatment cannot cure and survival time is likely to be short as is common in NSCLC the physical, emotional, philosophical and spiritual dimensions of the remaining life require the greatest consideration and sensitivity of assessment. For example, symptom control, quality of life or toxicity, convenience and financial costs of treatment could not be meaningfully evaluated in this meta-analysis by Sculier et al and it is important to consider these before totally discounting the role of MMC in advanced NSCLC. This realization about the meaningful end-points to be studied becomes even more apparent by reviewing large recent randomized trials performed by ECOG and the EORTC (Giaccone et al, 1998; Bonomi et al, 2000). These trials did not show any significant survival advantage between newer agents like paclitaxel or gemcitabine in combination with cisplatin when compared to the older chemotherapeutic regimes. Survival benefit has however been reported in studies using the combination of cisplatin and vinorelbine (Le Chevalier et al, 1996; Wozniak et al, 2000). Some treatment combinations such as gemcitabine and cisplatin yield equivalent or higher response rates and in some studies prolong time to disease progression (Evans et al, 1999). Hence it is important to consider all relevant end-points before discounting a drug purely on its inability to improve survival alone.

Health economic assessments of chemotherapy for advanced NSCLC have been conducted in the Canadian and UK healthcare systems. These have shown an economic advantage for patients treated with chemotherapy over the cost of patients who received supportive care alone (Jaakkimainen et al, 1990; Anderson et al, 2000). More recent work has shown significant differences in the costs associated with different regimens (Berthelot et al, 2000). The application of this analysis to other healthcare systems is uncertain but nonetheless cannot be ignored.

The symptomatic benefit associated with chemotherapy has been emphasized in several studies with overall relief of symptoms in greater than two-thirds of patients (Ellis et al, 1995). Quality of life should be an important component of clinical studies in advanced NSCLC and along with health economics would be helpful in deciding between different treatment options. Quality of life (QoL) in particular whilst mandatory in all cancer trials requires further assessment as to how much the results obtained by current techniques have actually influenced practice. Simplification of QoL techniques to improve clinical utility are to be encouraged. Any QoL instrument should be simple to administer and easy to explain, complete and analyse (Donnelly and Walsh, 1996).

This meta-analysis, which although it does not show an improvement in outcome in terms of survival as a primary end-point, should be viewed in the context of how we may better expand our knowledge base of new agents by phase III study. Importantly we should consider carefully what we want to achieve for our patients by different combinations of therapies. We would like to propose for initial discussion that

1. Eligibility for palliative studies should be unified to stage IV disease and IIIB with pleural effusion, i.e. patients for whom radical local therapy in particular with radiotherapy would not be contemplated.
2. There is consistency in reporting of 1-year survival as a primary end-point.
3. Quality of life scores should be performed using a straightforward validated instrument.

### REFERENCES

Anderson H, Hopwood P and Stephens RJ, et al (2000) Gemcitabine plus best supportive care (BSC) vs BSC in inoperable non-small cell lung cancer-a randomised trial with quality of life as the primary outcome. *Br J Cancer* **83**(4): 447–453

Bakowski MT and Crouch JC (1983) Chemotherapy of non-small cell lung cancer: a reappraisal and a look to the future. *Cancer Treat Rev* **10**: 159–172.

Berthelot JM, Will BP, Evans WK, Coyle D, Earle CC and Bordeleau L (2000). Decision framework for chemotherapeutic interventions for metastatic non-small cell lung cancer. *J Natl Cancer Inst* **92**(16): 1321–1329

Bonomi P, Kim K, Fairclough D and Cella D, et al (2000). Comparison of survival and quality of life in advanced non-small cell lung cancer patients treated with two dose levels of paclitaxel combined with cisplatin versus etoposide with cisplatin: results of an Eastern Cooperative Oncology Group trial. *J Clin Oncol* **18**(3): 623–631

Bunn PA (1996) New drug combinations in the treatment of advanced non-small cell (NSCLC) and small cell (SCLC) lung cancer. In: American Society of Clinical Oncology educational book p204.

Cullen MH, Woodroffe CM and Billingham LJ, et al (1999). Mitomycin, ifosfamide and cisplatin in unresectable non-small cell lung cancer: effects on survival and quality of life. *J Clin Oncol* **17**(10): 3188–3194

Donnelly S and Walsh D (1996) Quality of life assessment in advanced cancer. *Palliat Med* **10**: 275–283

Ellis PA, Smith IE, Hardy JR, Nicolson MC, Talbot DC, Ashley SE and Pricst K (1995) Symptom relief with MVP (mitomycin C, vinblastine and cisplatin) chemotherapy in advanced non-small cell lung cancer. *Br J Cancer* **71**(2): 366–370

Evans WK, Kocha W, Gagliardi A, Eady A and Newman TE (1999) The use of gemcitabine in non-small cell lung cancer. Provincial Lung Cancer Disease Site Group. Provincial Systemic Treatment Disease Site Group. *Cancer Prev Contrl* **3**(1): 84–94

Giaccone G, Splinter TA, Debruyne C and Kho GS, et al (1998) Randomized study of paclitaxel-cisplatin versus cisplatin-teniposide in patients with advanced non-small cell lung cancer. The European Organization for Research and Treatment of Cancer, Lung Cancer Cooperative Group. *J Clin Oncol* **16**(6): 2133–2141

Jaakkimainen L, Goodwin PJ, Pater J, Warde P, Murray N and Rapp E for the NCI clinical trials group (1990). Counting the costs of chemotherapy in randomised trials in non-small cell lung cancer. *J Clin Oncol* **8**(8): 1301–1309

Le Chevalier T, Brisgand D, Pujol JL and Douillard JY, et al (1996) Results of a randomized study comparing combination of navelbine-cisplatin to combination of vindesine-cisplatin and to navelbine alone in 612 patients with inoperable non-small cell lung cancer. *Bull Cancer* **83**(5): 385–394

Luedke DW, Einhorn L, Omura GA, Sarma PR, Bartolucci AA, Birch R and Greco FA (1990) Randomized comparison of two combination regimens versus minimal chemotherapy in non-small cell lung cancer: a Southeastern Cancer Study Group Trial. *J Clin Oncol* **8**: 886–891

Non-small Cell Lung Cancer Collaborative Group (1995) Chemotherapy in non-small cell lung cancer: a meta-analysis using updated data on

 *© 2001 Cancer Research Campaign*

individual patients from 52 randomised clinical trials. *Br Med J* **311**: 899–909
Rosell R, Gomez-Codina J, Camps C and Maestre J, et al (1994) A randomized trial comparing preoperative chemotherapy plus surgery with surgery alone in patients with non-small cell lung cancer. *New Eng J Med* **330**: 153–158
Roth JA, Fossell F, Komaki R and Ryan MB, et al (1994) A randomized trial comparing perioperative chemotherapy and surgery with surgery alone in resectable stage IIIA non-small cell lung cancer. *J Natl Cancer Inst* **86**: 673–680
Sculier JP, Ghisdal L, Berghamans T and Branle F, et al (2001). The role of Mitomycin C in the treatment of Non-Small Cell Lung Cancer: A systemic review with meta-analysis of the literature. *Br J Cancer*
Wozniak AJ, Crowley JJ, Balcerzak SP and Weiss GR, et al (1998) Randomized trial comparing cisplatin with cisplatin plus vinorelbine in the treatment of advanced non-small cell lung cancer: a Southwest Oncology Group study. *J Clin Oncol* **16**(7): 2459–2465

*© 2001 Cancer Research Campaign* 

